# Non-Hormonal Contraception

**DOI:** 10.3390/jcm12144791

**Published:** 2023-07-20

**Authors:** Sarah Anne Howard, Soumya Rahima Benhabbour

**Affiliations:** 1 Division of Pharmacoengineering and Molecular Pharmaceutics, UNC Eshelman School of Pharmacy, University of North Carolina at Chapel Hill, Chapel Hill, NC 27599, USA; sah@email.unc.edu; 2 Joint Department of Biomedical Engineering, North Carolina State University and The University of North Carolina at Chapel Hill, Chapel Hill, NC 27599, USA

**Keywords:** contraceptive, non-hormonal, long-acting, reversible

## Abstract

While hormonal contraceptives are efficacious and available in several forms for women, perception of safety and concern over side effects are a deterrent for many. Existing non-hormonal contraceptives include permanent sterilization, copper intrauterine devices (IUDs), chemical/physical barriers such as spermicides and condoms, as well as traditional family planning methods including withdrawal and the rhythm method. Individuals who wish to retain their fertility in the future can achieve highest adherence and efficacy with long-acting, reversible contraceptives (LARCs), though there is only one, the copper IUD, that is non-hormonal. As rates of unintended pregnancies remain high with existing contraceptive options, it is becoming increasingly attractive to develop novel pregnancy prevention methods for both women and men. Non-hormonal contraceptives can target a variety of critical reproductive processes discussed here. This review focuses on identified non-hormonal contraceptive targets and subsequent drug candidates in development.

## 1. Introduction

### 1.1. History of Contraceptives

The need for family planning is engraved in the history of humankind.

The Kahun Papyrus, believed to be written in Egypt circa 1825 BCE, describes early gynecological practices in hieroglyphics—including a recipe for a contraceptive suppository utilizing crocodile feces. Approximately 300 years later, the Ebers Medical Papyrus presented an herbal contraceptive suppository recipe with honey and acacia [1]. Acacia, upon fermentation, can produce lactic acid anhydride which may have been effective through vaginal pH modulation. While the use of plant-based suppositories was continued by the Ancient Greeks, they also explored oral herbal concoctions and early calendar-based family planning [2,3].

Additionally, penile sheaths have been described throughout history, though it is suggested their original purpose was to prevent transmission of disease rather than conception. These precursors to modern condoms were often made of cloth or animal organs [4,5]. The popularity of these sheaths increased during the Renaissance among the affluent until religious officials began to notice an associated population decline, henceforth declaring their use as a ‘sin’ [5].

In the 19th and early 20th centuries, the world saw major advances in contraceptive science. The invention of vulcanized rubber, and eventually latex, led to the widespread production and popularity of the condom. Additionally, scientific research on the female reproductive system soared, leading to the discovery of the mammalian ovum and reproductive hormones [6]. Western attitudes on reproduction and contraception were mixed at this time, with obscenity laws often preventing the sale or use of contraceptives [7]. Rather, initial medical uses of reproductive hormones were to treat gynecological disorders, such as dysmenorrhea.

Contraceptive options began expanding in the 1950s when the spermicide Nonoxynol-9 was introduced, and in 1960 the United States approved its first contraceptive pill, Enovid [8]. Each dose of “The Pill”, as it came to be known, contained 0.15 mg of a synthetic estradiol (mestranol) and 10 mg of a synthetic progestin (noretynodrel) to inhibit ovulation. Cardiovascular concerns eventually led to the discontinuation of high-estrogen combined oral contraceptives (COCs), which have since been replaced with low- and ultra-low dose COC options as well as microdose progestin-only pills (POPs or minipills) [9]. Moreover, while surgical methods for permanent sterilization have existed since 1880, substantial utilization in the United States did not begin until the 1970s [10].

While the decades since have welcomed many new contraceptive technologies, like the intravaginal ring or contraceptive implant, only the copper IUD and on-demand barrier methods offer reversible non-hormonal contraception. Furthermore, a significant lack of male-controlled options currently prevents men from accessing highly efficacious, yet reversible, control over their reproduction [11]. While investigative male hormonal contraceptives using testosterone and its synthetic analogs have been developed and reached clinical trials, none have obtained FDA approval [12].

### 1.2. Overview of Human Reproduction

Hormonal contraceptives, by their nature, prevent pregnancy by regulating pituitary production of follicle-stimulating hormone (FSH) and luteinizing hormone (LH), which act as reproductive signals for ovulation in women and sperm maturation in men [13,14]. Additional effects of endogenous hormones include thinning of the uterine endometrium and thickening of cervical mucous, which may play a role in preventing pregnancy [15]. In contrast, non-hormonal alternatives are not restricted to targeting the hypothalamic–pituitary–gonadal axis and can instead impact various stages of reproduction. Broadly, these processes include gametogenesis and fertilization/implantation. Diploid primordial germ cells are the origin of all human gametes, though the process of gametogenesis occurs differently between the sexes [16].

**Figure 1:**
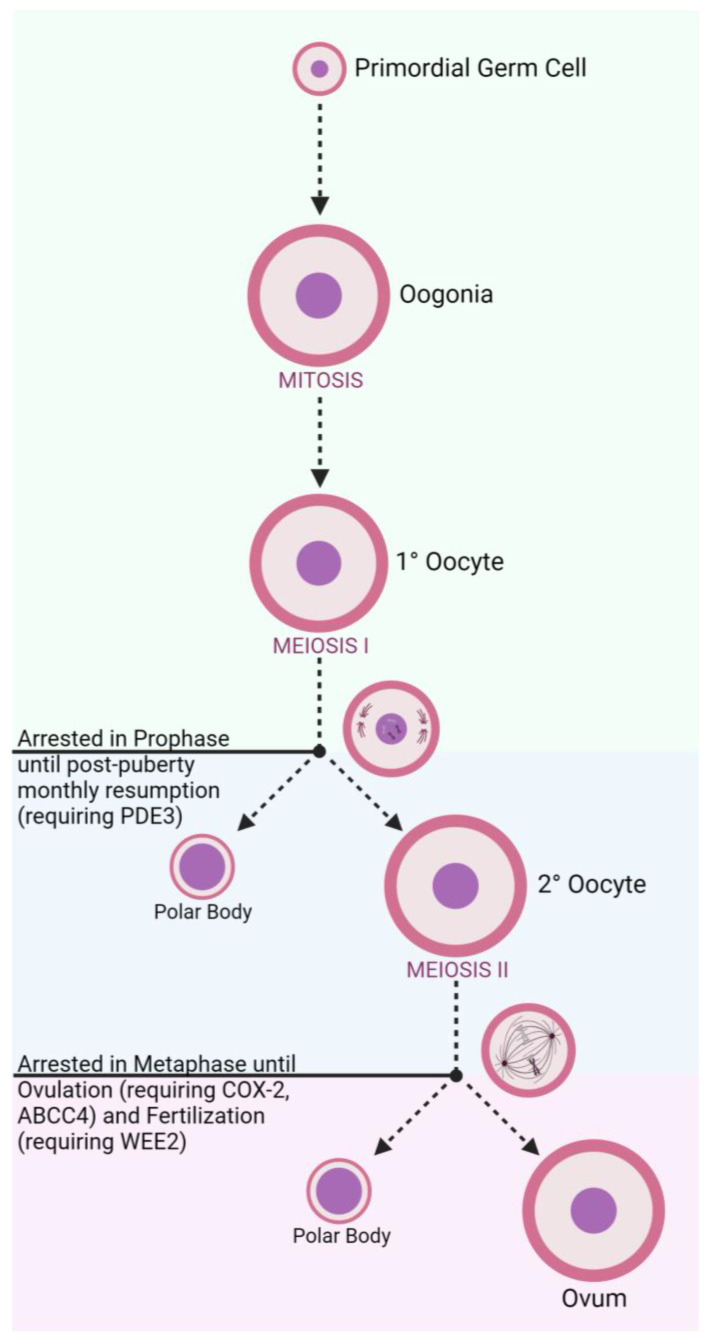
Oogenesis and Stage-Related Contraceptive Targets.

In developing female fetuses (Figure 1), oogonia divide mitotically to create primary oocytes. From there, meiosis begins to create secondary oocytes in the ovary, though this process is halted during prophase until puberty is reached and cascading effects of menstrual LH surges continue the meiotic process [17]. A secondary oocyte develops into a fully matured ovum following an additional meiotic division, though this process is arrested during metaphase until the oocyte is ovulated and fertilized [17]. These cell division steps also produce diminutive byproduct cells known as polar bodies that are later degraded. Concurrent development of follicular cells generates protective layers to encapsulate the oocyte, including the zona pellucida (ZP) which surrounds the oocyte plasma membrane [18].

**Figure 2:**
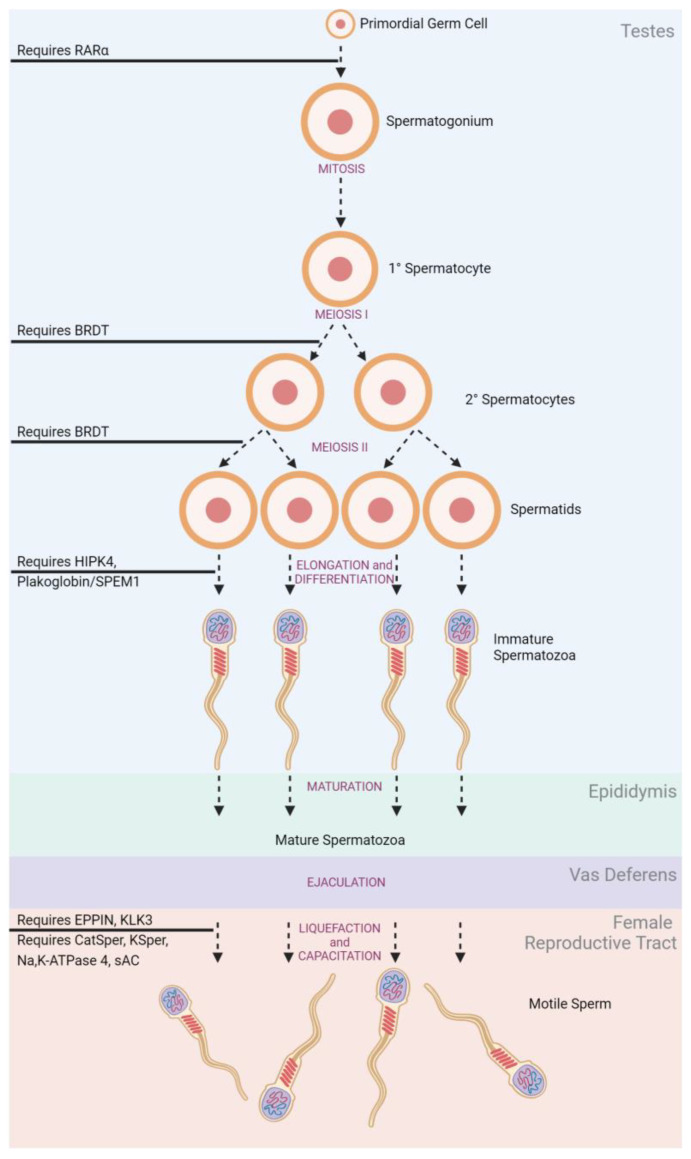
Spermatogenesis and Stage-Related Contraceptive Targets.

In adult males (Figure 2), spermatogonia mature under mitotic division within the seminiferous tubules of the testes [19]. The structure of the seminiferous tubules is supported by epithelial Sertoli cells which create an immunological barrier to protect sperm, known as the blood–testes–barrier (BTB) [20,21]. Produced spermatocytes remain in the seminiferous tubules and undergo a series of successive meiotic divisions to develop into spermatids. Spermatids remain anchored to Sertoli cells for continued maturing into competent spermatozoa through elongation and differentiation [21]. Spermatozoa must be produced in large numbers (sperm count) with appropriate morphology and motile function to be effective at fertilization [22]. Upon ejaculation, the spermatozoa are ‘activated’ as they travel through the epididymis and gain their progressive motility [23].

**Figure 3:**
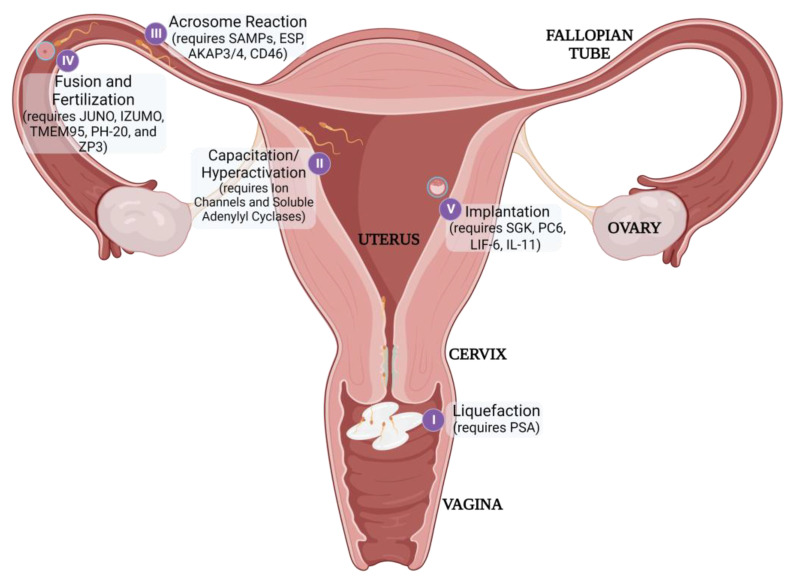
Post-gametogenesis Reproductive Processes and Stage-Related Contraceptive Targets (table in Section 3.2).

Fertilization itself requires sperm to traverse the vagina, cervix, and uterus to reach the oocyte in the fallopian tube (Figure 3). The viscosity of the cervical mucus serves as an initial barrier and filters out poor-quality sperm. Additionally, the natural pH of the vaginal environment is too acidic to support sperm viability [24]. Therefore, sperm is transported in seminal fluid that can buffer the vaginal pH to achieve an acceptable, neutral pH. Once sperm has entered the female reproductive tract, it must undergo capacitation to achieve the additional physical characteristics necessary to penetrate the protective ZP, including liquefaction, hyperactivation, and acrosomal reactiveness [25]. Immediately after ejaculation, seminal proteins (SEMG1/2) coagulate and encapsulate spermatozoa. This gelation inhibits the progression of sperm, so prostate-specific antigen (PSA) secreted during ejaculation must subsequently liquefy the matrix to release the motile sperm [26]. Henceforth, the sperm must then achieve hyperactive motility to efficiently move through the fallopian tubes and penetrate the ZP [27]. Lastly, spermatozoa need to be prepared to undergo the ‘Acrosome Reaction’ when nearing the oocyte. This reaction releases enzymes that can help degrade the ZP to further enhance penetration as well as exposing egg-binding proteins to facilitate fusion between the gametes [28].

Once fertilization has occurred, the rapidly dividing embryo must then implant successfully in the uterus to continue developing [29]. Each distinct reproductive process may serve as a potential target for non-hormonal contraception.

### 1.3. Unintended Pregnancy

Unintended pregnancies are pregnancies that occur in advance of a preferred timeframe or completely unplanned. Each year, 121 million unintended pregnancies occur, accounting for nearly half (48%) of all pregnancies across the globe. In low- and middle-income countries, the rate of unintended pregnancies averages 93% and 66%, respectively [30].

When compared to children born of planned pregnancies, children born as a result of an unintended pregnancy are more likely to be premature, of low-birth weight, and be breastfed for a shorter length of time, or not at all [31,32,33]. These children may also be at risk of developmental delays that can impact their long-term social, emotional, and academic success [32]. The mothers are also at increased risk of pregnancy unhappiness, post-partum depression, and maternal mortality [34].

Over 60% of unintended pregnancies result in abortion, with no difference in prevalence among countries with or without abortion restrictions [30]. Furthermore, 45% of these abortions are performed in unsafe conditions, without a proper method or without a trained professional [35,36]. A majority of these occur in developing countries, where 6.9 million women each year receive medical treatment as a result of an unsafe abortion [37].

Contraceptives offer robust solutions to unintended pregnancy and its associated outcomes, with models predicting that expanded access and use of contraceptives may result in up to a 33% reduction in maternal mortality rates [38]. In this review, existing non-hormonal contraceptives, those currently in development, and potential targets for prevention of pregnancy will be discussed.

## 2. Current Solutions

Individual attitudes and values toward reproduction lead many to consider contraceptives for preventing, or managing the timing of, pregnancy [39]. The contraceptive methods employed today encompass hormonal methods, like the oral contraceptive pill and intravaginal ring, as well as non-hormonal methods, like sterilization and natural family planning, alike. Natural, or traditional, contraception options like the calendar method do not require surgery or pharmaceuticals, yet they are less prevalent than ‘modern’ methods and are utilized by only 4% of women of reproductive age [40]. In fact, the latest update from the United Nations shows that female sterilization, or tubal ligation, is used by 24% of women, making it the single most common contraceptive method in use across the globe—though this is skewed by the large utilization rate among women aged 30 years and above. Male sterilization, on the other hand, is the primary tool for pregnancy prevention for only 2% of women. The second most common contraceptive method is the male condom, with its ease of access and use, making it the preferred choice for over one-fifth of contraceptive-utilizing women. In total, all hormonal methods combined, including the oral pill, intrauterine devices, implants, and injectables, make up nearly 40% of all contraceptive use. Still, 17% of women who desire to control their reproduction do not have their needs met by existing options and further yet 24% of women are unsatisfied with modern methods [40].

It is important to note the various influences that affect a person’s contraceptive method selection [41,42]. Many have their options limited by accessibility, with contraceptive use prohibited by direct barriers like legislative constraints [43,44] or high costs [45] Additional indirect barriers such as stigmatization and lack of education also reduce utilization of highly efficacious contraceptives [46,47]. Misconceptions and anecdotes regarding the safety and side-effects of modern contraceptives are prevalent globally and can affect uptake and adherence [48]. Cultural differences can also alter the influence that certain barriers have over contraceptive use. For example, a study across several urban African countries observed that communities with more women believing in contraceptive myths, such as the statement “people who use contraceptives end up with health problems”, had increased contraceptive use. In contrast, the communities where men firmly believed in these myths had decreased utilization [48].

Characteristics of individual methods are important for users to consider when selecting a preferred contraceptive option, including efficacy, route of administration, convenience, partner cooperation, and side effects [41]. Hormonal contraceptives have long been associated with several unpleasant side effects, such as weight gain, nausea, mood instability, and more. In fact, the side effects of oral contraceptive pills are most commonly cited as reason for discontinuation, and nearly two-fifths of women may actually perceive them to be ‘unsafe’ [49]. Therefore, it is abundantly clear that a large demand exists for non-hormonal contraceptives, but the scarcity of existing options has left an unmet need.

### 2.1. Existing Non-Hormonal Contraceptives

For sexually active individuals who wish to avoid hormonal contraceptives, a handful of options with varying efficacies and benefits exist (Table 1).

#### 2.1.1. Permanent Non-Hormonal Contraceptives

Permanent sterilizations, including tubal ligations and vasectomies, are medical procedures intended to irreversibly inhibit fertility. They are among the most efficacious of the non-hormonal contraceptive methods [57]. Failure rates are further reduced when both members of a couple have undergone surgical sterilization, though decisions must be made carefully due to their permanent nature.

1. Female Sterilization

The long-lasting effects of female sterilization are desired by many, with a quarter of all women in the United States utilizing it as their primary contraceptive, though prevalence is skewed by increased implementation among woman over the age of 40 [58]. Advantages of female sterilization include elimination of the need for contraceptive regimen adherence and lack of dependence on partner compliance [59].

Tubal ligations typically utilize laparoscopic techniques to surgically access then occlude, or resect, both fallopian tubes. This procedure prevents pregnancy by preventing released eggs from reaching the ovaries while also physically blocking sperm from accessing the egg. In the United States these procedures are often performed under general anesthesia, but global utilization of local anesthetic is increasing [60,61]. Local anesthetic use is also prevalent in sterilizations performed using modernly developed hysteroscopic techniques due to their reduced invasiveness. With this alternative, transcervical bilateral tubal occlusion is achieved through placement of metal microinserts containing polyethylene terephthalate (PET) fibers that induce local fibrotic growth over the twelve weeks following the procedure [62].

Given the convenience, many women choose to undergo tubal ligations following a vaginal or cesarean delivery, known as a postpartum ligation. Recent research suggests, though, that only half of American women who desire a postpartum ligation receive one [63]. This is partially attributed to state and federal requirements for anyone seeking permanent sterilization to undergo a thirty-day waiting, or reflection, period following signature of a consent form [64]. Nulliparous women, those who have never given birth, and young women also experience difficulties accessing desired sterilization. Common physician-imposed restrictions are reported to include age limits, minimums for existing children, and spousal consent [65].

On average, one out of every ten women who undergo sterilization in the US experience sterilization regret, though rates decline as age upon procedure increases [59,66,67].

2. Male Sterilization

In contrast, males who undergo sterilization experience regret at a much lower rate, only about 5% [59,67]. Vasectomies are typically considered to be easier to perform and less invasive than tubal ligations, requiring only a 15-min outpatient procedure with local anesthetic. For decades, vasectomy required scrotal incision to access the vas deferens for occlusion, but recent advancements have popularized the no-scalpel vasectomy that requires only a single puncture and eliminates the need for healing sutures [68]. Once reached, the vas deferens can be occluded through a variety of techniques, though excision of a segment of the vas and ligation with sutures are the most popular. While reversal of these methods is possible through vasovasostomy or vasoepididymostomy, the success of reversal declines as the length of time since vasectomy increases [69]. Alternatively, sperm can be retrieved through percutaneous epidydimal, or testicular, aspiration and extraction for intracytoplasmic sperm injection combined with in vitro fertilization rather than reversing vasectomy. While this option can be less expensive for the male partner, the monetary and physical costs of IVF for the female partner may be prohibitive.

#### 2.1.2. Reversible Non-Hormonal Contraceptives

Reversible contraceptives offer temporary prevention of pregnancy for users who may wish to conceive in the future or simply those who want to avoid an invasive surgery. These methods can be long-acting, like the intrauterine device (IUD) and contraceptive implant, or short-acting like barrier methods or the hormonal daily pill. While hormonal options are the most prevalent reversible contraception option, interest in non-hormonal alternatives continues to grow, especially for those wishing to reduce side-effects of contraceptive use. Though efficacy and ease of adherence are additionally important factors in contraceptive selection, the only long-acting reversible non-hormonal option with greater than 99% efficacy is the copper IUD.

1. Copper Intrauterine Device

Additional modern non-hormonal methods are temporary, with the copper IUD as the sole long-acting option. The copper IUD, first approved by the FDA in 1984 under the brand name ParaGard, generally consists of a polyethylene ‘T’ wrapped with copper wire. ParaGard contains 380 mm^2^ of copper and is approved for 5–10 years of use, though discontinuation often occurs in the first year due to increased or abnormal bleeding and pelvic pain [70,71,72]. Variations of the traditional copper IUD design, such as the Ballerine intrauterine ball, have also been produced to improve the user experience [73]. The mechanism-of-action for the copper IUD is not entirely understood, though prevailing research suggests that copper ions released from the IUD cause a localized inflammatory response in the uterine cavity that inhibits sperm motility and survival. This effect happens rapidly, and users can rely on the copper IUD immediately following insertion. This also allows the copper IUD to be utilized for emergency contraception (EC), working to prevent implantation for up to 5 days following unprotected intercourse. Hormonal ECs, containing levonorgestrel, can have reduced efficacy in users with a body mass index (BMI) over 30 [74]. Overall, the copper IUD is ten times more effective than hormonal ECs and does not have efficacy differences as a result of user BMI; however, both insertion and removal require a trained physician [53].

2. Chemical and Physical Barriers

Spermicides are one of the least effective forms of modern contraceptives. Available formulations possess between 70–80% efficacy with typical use [54,75]. Spermicides, functioning as chemical barriers to fertilization, contain active ingredients inhospitable to sperm. While available in several different administration forms such as creams, gels, films, and suppositories, traditional spermicides in the United States all function through the active ingredient Nonoxynol-9 (N9). N9 is a nonionic surfactant that disrupts the acrosomal and midpiece membranes of sperm, leading to sperm immobilization and, inevitably, sperm death [76].

The recently approved contraceptive gel, Phexxi, also provides a chemical barrier to conception by buffering the vaginal environment to maintain an acidic pH. It is currently available via prescription and is the only topical contraceptive alternative to N9 approved in the US [77]. With typical use, Phexxi is 86% effective at preventing pregnancy.

Lastly, physical barrier methods are the only approved modern contraception technologies that can also provide protection against sexually transmitted infections, or STIs [78]. Barrier methods include condoms, diaphragms, sponges, and cervical caps. The male condom, an externally-worn sheath typically made of latex, is the second-most common form of contraception used across the globe, surpassed only by permanent sterilization [40], likely due to its ease of use, low cost, and high accessibility [79] Condoms are often available with lubricants and spermicidal coatings, which can enhance the overall efficiency [80]. Combined with a perception of reduced pleasure and a dependence on strict adherence, like many short-acting contraceptive methods, male condoms have a 15% failure rate with typical use [81].

3. Traditional Family Planning

Approximately 19 million women rely on ‘traditional’ contraceptive methods. Traditional contraceptive options include withdrawal and fertility tracking [82,83]. Withdrawal, or coitus interruptus, consists of halting intercourse prior to ejaculation. Pre-ejaculatory fluids and human error can make this method unreliable, with one out of every five women using this method likely to fall pregnant within a year [56]. Fertility tracking includes the calendar, or rhythm method, temperature tracking, and the Billings method. Of the three, the calendar method is the most prevalently utilized [83] and requires several months of detailed tracking of menstrual cycles to calculate a woman’s likely ‘fertile phase.’ Fertility is also assessed through the temperature method, which asks users to check basal body temperatures every morning, and the Billings method, which has users compare vaginal mucus elasticity over time. While the efficacy of these methods is typically lower than modern alternatives, these options are highly valued by women with medical, cultural, or religious objections to modern methods [84].

Overall, the only currently available non-hormonal contraceptive method that is highly efficacious with typical use is the copper IUD. Ongoing research continues to develop new, efficacious, and acceptable alternatives for use by both women and men.

## 3. Non-Hormonal Contraceptives in Development

Significant efforts are underway to develop new non-hormonal contraceptives. The 2020 approval of Phexxi as the first topical contraceptive to compete with nonoxynol-9 and other ongoing clinical trials may indicate a scientific resurgence in non-hormonal contraception exploration. Ongoing research to identify novel contraceptive targets and develop non-hormonal therapeutics encompass four main categories: Locally Acting Microbials and Spermicides, Reproductive Protein Inhibition, Immunocontraception, and Non-Surgical Alternatives for Permanent Sterilization.

### 3.1. Locally Acting Microbials and Spermicides

With growing concern about the safety and efficacy of the spermicide nonoxynol-9 [85,86], it is imperative to develop additional antimicrobial and spermicidal alternatives. While Phexxi has capitalized on the contraceptive gap by delivering lactic acid, citric acid, and potassium bitartrate as an intravaginal gel, other spermiostatic and spermicidal substances are being actively explored. Additionally, some antimicrobials may show efficacy in preventing sexually-transmitted infections, making them especially of interest for multipurpose prevention technology (MPT) development [87].

Much like Phexxi, Dare Bioscience’s Ovaprene combines several actives for a multiple-modality approach to contraception. Ovaprene is a monthly silicone intravaginal ring with a knitted polymer mesh filling the middle of the ring to provide a physical barrier to sperm while delivering ferrous gluconate and ascorbic acid to reduce sperm motility [88]. It has completed Phase II clinical trials (NCT03598088) in the United States and has recently received an Investigational Device Exemption to begin Phase III studies soon. Upon approval, Ovaprene could be the first reversible, yet long-acting, user-controlled non-hormonal contraceptive; however, concerns exist, as 24% of women utilizing the device in Phase II experienced bacterial vaginosis (BV). BV is a vaginal microbiome imbalance with symptoms including vaginal discharge and malodor that can increase a woman’s risk of contracting an STI or other infection [89]. In contrast, the same number of women utilizing an on-demand diaphragm for contraception had zero incidences of BV during the study period.

UniPron is another antimicrobial vaginal gel in development. Utilizing citric acid to maintain an acidic vaginal environment, it has been shown to be safe and efficacious in non-human primates [90,91]. Additional studies have been proposed to explore the ability of Unipron, and citric acid, to combat STIs [92].

The polymeric styrene maleic anhydride (SMA), used in the intravasal male contraceptive hydrogel RISUG (Reversible Inhibition of Sperm Under Guidance), may also work by modifying local pH [93]. While the driving spermicidal action of SMA is unclear, several mechanisms have been proposed, including pH lowering that may be caused by positively charged precipitates that interact with sperm during ejaculation [94]. SMA also provides partial occlusion, as the polymer hydrolyzes after injection into the vas deferens. Though RISUG, having completed clinical trials in India [95], is marketed as a less invasive alternative to vasectomy, pre-clinical animal studies show that RISUG may be reversible through dissolution of the polymer. A cuproferro-composite of SMA, called Smart RISUG, is actively being developed and studied to provide better detectability and contraceptive control than its predecessor [96]. In the United States, the RISUG technology has been licensed and development of another intravasal injectable SMA contraceptive is being developed under the name Vasalgel. Research indicates that nearly one-third of male college students are interested in using the technology, while others had uncertainties—most often due to novelty and the route of administration [97]. Preliminary work has also begun to utilize an SMA-hydrogel as an occlusive female contraceptive through implantation in the fallopian tubes [98].

Polystyrene sulfonate (PSS) has shown efficacy in a Phase I trial as the active ingredient in a female contraceptive gel [99]. Currently approved to treat elevated potassium in plasma, PSS has been shown to inhibit several sperm-related enzymes critical to fertilization, such as acrosin and hyaluronidase. Furthermore, it possesses antimicrobial properties against several STIs, including HIV-1 and chlamydia [100]. In 2005, PSS was formulated into an intravaginal film, but no further studies have been published [101].

A variety of naturally-derived antimicrobials have also been studied for their contraceptive benefits, though none have progressed beyond pre-clinical studies. Magainin, isolated from frog mucosal secretions, and Nisin, a product of *Lactococcus lactis*, found in dairy products, are antimicrobial peptides (AMPs) that possess both antipathogenic and spermicidal or spermiostatic properties, respectively [87].

Curcumin, found in turmeric, can significantly reduce sperm motility and function upon exposure to high concentrations [102]. Its broad antimicrobial benefits and general high tolerability inspire potential for additional therapeutic and contraceptive use [103].

Polymeric nanomesh fibers loaded with glycerol monolaurate (GML, or monolaurin), found in coconut oil, have shown in vitro activity against both HIV-1 and sperm [104]. It is hypothesized that GML may embed itself into lipid membranes and inhibit signal transduction.

Lupeol is another potential contraceptive antimicrobial with an unclear mechanism of action. A triterpenoid, Lupeol is found in a variety of fruits and vegetables and its anti-inflammatory benefits have been well documented [105]. Lupeol has been observed to affect sperm hyperactivation, a stage of enhanced sperm motility necessary for egg fertilization, though the source of this effect is contested. It was originally believed that Lupeol inhibited hyperactivation by binding to ABHD2 and obstructing activation of the sperm calcium channel CatSper [106], but attempts to replicate this research have been unsuccessful [107].

### 3.2. Small Molecule Reproductive Protein Inhibition

Various proteins and enzymes at each phase of human reproduction have been identified as potential contraceptive targets (Table 2).

#### 3.2.1. Disruption of Gamete Production

Spermatogenesis (Figure 2) is the continuous sequence of cell proliferation and differentiation by which germ cells become spermatozoa. Following the onset of puberty, spermatogenesis occurs continuously with millions of sperm produced daily [153]. This makes proteins relevant to spermatogenesis extremely interesting for reversible male contraception, as their cessation should allow for spermatogenesis, and therefore fertility, to rapidly resume [154]. Several proteins have been established as fundamental to this phase of sperm production.

Testis-specific bromodomain, BRDT, is an epigenetic reader protein expressed in male germ cells. Inhibition of BRDT hinders chromatin remodeling, which leads to spermatozoa developing with irregular morphology and motility [108]. The effects of a small-molecule inhibitor called JQ1 were studied, and complete, yet reversible, contraception in mice was observed. JQ1 has not been developed further, as it binds off-target to transcription-regulating bromodomain reader proteins throughout the body. Additionally, JQ1 was shown to rapidly clear (t_1/2_ = 1–2 h) from system circulation when administered orally or intravenously to mice, further limiting its potential by requiring exceedingly frequent doses [109,110]. The development of JQ1 highlighted the potential of targeting BRDT for contraception, though, and new highly specific inhibitors are currently being developed and screened for contraceptive potential [155].

The nuclear Retinoic Acid Receptors (RARs) are male contraceptive targets because Retinoic Acid (RA) has an important role in several male reproductive processes, including spermatogenic differentiation [156]. Male mice with RARα genetic knockout are completely infertile with no observed side-effects. Several small molecule antagonists have been screened for selection against RAR, with the recent discovery of YCT529 garnering attention due to its contraceptive success following oral administration in mice [111].

Homeodomain-interacting protein kinase-4 (HIPK4) is expressed in spermatids and is essential for spermatozoa to develop the elongated phenotype [112]. The family of testis-specific serine/threonine kinases (TSSKs) are also necessary components for spermatogenic differentiation [113]. Additional protein kinases crucial to sperm formation and maturation have also been identified, though specific drug candidates are yet to be developed.

Many drug candidates that inhibit spermatogenesis through a multitude of protein targets have also been identified, several of which are naturally derived. Oleanolic acid, a triterpenoid compound found in many plants, has long been known to reduce fertility and sperm motility in male rats [114,115,116]. The mechanism by which this occurs was only recently determined to be due to disruption of inter-sertoli junctions and resulting increases in blood-testes barrier permeability, which can expose immature sperm to toxic agents [117].

The antioxidant β-caryophyllene, which contributes to the spice of black pepper and cloves, is also an agonist of type 2 cannabinoid receptors (CBR2). These receptors are commonly expressed in immune-related cells during active inflammation as well as in testicular germ cells. Male rats treated with β-caryophyllene had reduced sperm motility [121], abnormal sperm morphology, and reduced total sperm count [118].

Elongating and elongated spermatids anchor themselves to Sertoli cells, the somatic cells of the testicular epithelium that offer support during spermatogenesis, for stability during maturation. Disrupting these interactions can cause untimely shedding of germ cells and subsequent male infertility. The herb-derived Triptonide binds to plakoglobin, which mediates desmosomal junctions between Sertoli cells and between Sertoli cells and germ cells. This binding is believed to inhibit the essential interaction between desmosomal plakoglobin and its partner, SPEM 1, exclusively found in elongating and elongated spermatids. Thus, Triptonide may impede nucleocytoplasmic transport, resulting in abnormal sperm morphology. Non-human primate studies with daily oral Triptonide showed contraceptive efficacy for over two years without observation of side-effects [119] Additionally, sperm morphology and function were restored within weeks of treatment cessation, making Triptonide an especially attractive compound for reversible male contraception [119].

Lonidamide analogs, such as Adjudin [120] and H2-Gamendazole, have also been observed to interfere with Sertoli–germ cell junctions [121]. While safety concerns have limited their development, next-generation derivatives are being explored [157].

Phosphodiesterase in the female reproductive system may offer a unique pathway to contraception via disruption of final oocyte maturation prior to ovulation [158]. Phosphodiesterase 3A (PDE3) located in the oocyte prevents accumulation of cyclic adenosine monophosphate (cAMP). Inhibition of PDE3 prevents cAMP degradation, which inhibits meiotic resumption, rendering monthly ovulated oocytes as immature and non-fertilizable. Inhibitors have been shown to maintain this meiotic arrest in vitro, such as in the commercially available Milrinone. Milrinone is currently utilized for pulmonary vasodilation due to the expression of PDE3 in cardiac myocytes. This cross-reactivity is a concern, requiring the development of inhibitors with increased specificity. One example, ORG9935, has been developed as a potential female contraception, and its (-)-enantiomer, ORG20864, preferentially binds to oocyte PDE3 over cardiac PDE3 in mice [123].

#### 3.2.2. Disruption of Sperm Transit

While proper development of spermatozoa is a crucial first step for reproduction, sperm motility must be maintained to reach and fertilize an oocyte. Motility can be reduced in otherwise healthy sperm by targeting proteins necessary for sperm capacitation and hyperactivation.

The sperm-surface protein EPPIN, or epididymal protease inhibitor, plays an important role in sperm motility [159]. As EPPIN briefly binds with the protein SEMG1 found in semen, it institutes a temporary loss of sperm motility by gelation. PSA typically reverses this through semen liquefaction, but the investigative, and highly selective, drug compound EP055 irreversibly binds with EPPIN and prevents liquefaction. Intravenous administration of the drug has resulted in reversible contraception in male macaque studies [124]. PSA, sometimes known as KLK3, has also been inhibited through vaginal administration of AEBSF [125]. This safely prevented semen liquefaction in mice, but the compound showed human endocervical cell toxicity.

Several ion-transport mechanisms have significant effects on sperm motility. Na,K-ATPase4 [126], KSper, and CatSper are three sperm-specific ion channels that have been identified as potential male contraceptive targets given their role in sperm capacitation and hyperactivation [127]. While development of drug candidates for Na,K-ATPase4 and Ksper is in preliminary stages, an investigative CatSper inhibitor HC-056456 has been created [128].

Sperm capacitation proceeding ejaculation further relies on activation of soluble adenylyl cyclase isoforms, sACl and sACfl, not found in somatic tissues [129]. This has aided in the development of highly-specific inhibitors with potential for both male and female-controlled contraception.

#### 3.2.3. Disruption of Fertilization and Implantation

Lastly, various protein targets have been identified to interfere with the latter stages of reproduction including oocyte ovulation, fertilization, and subsequent embryo implantation.

Although contraceptive targets to directly disrupt oogenesis are extremely limited, non-hormonal targets to disrupt ovulation are more promising. Cyclooxygenase-2 (COX-2) in non-reproductive tissues is typically only found during acute pain and/or inflammation. Within the female reproductive system, though, it regulates prostaglandin synthesis and subsequent oocyte release through production of prostaglandin E2 [132] Both enzymes have been studied as potential targets for non-hormonal female contraception. Commercial COX-2 inhibitors, such as Celebrex and Meloxicam, have shown inhibition of ovulation in non-human primates [130], which showed efficacy as Emergency Contraception (EC) but not primary birth control. However, a clinical trial (NCT01129245) revealed that Celebrex did not have substantial potential as an EC in humans. In contrast, twice-daily administration of BAY06, a prostaglandin antagonist, resulted in substantial, yet reversible, decreases in fertility [131]—though no additional research on the compound has been published. ATP Binding Cassette Subfamily C Member 4 (ABCC4) has recently been found to be an important prostaglandin transporter and is upregulated in preparation for ovulation. ABCC4 may be another contraceptive target for inhibition of ovulation by reducing necessary prostaglandin efflux [132].

The oocyte-specific kinase WEE2 is a key player in the final meiotic resumption and overall oocyte maturation during fertilization, as first evidenced in genetic studies [133]. This has been further supported through case studies of infertile women with homozygous WEE2 mutations and phenotypic rescue following intraoocyte injection of complementary WEE2 RNA [133] Preliminary development of WEE2-selective inhibitors identified with high-throughput screening is underway [134].

Sperm and oocyte membrane fusion is pivotal to fertilization. A handful of key fusion proteins, including JUNO, IZUMO1, and TMEM95 [137] have been identified. IZUMO and JUNO are part of a ligand-receptor protein pair between the sperm and egg, respectively [160]. JUNO has high sequence conservation across mammals and has been the focus of small-molecule development to interfere with the overall JUNO/IZUMO complex [135].

Knock-out genetic studies have ascertained proteins specifically responsible for embryonic implantation that may lead to development of female non-hormonal contraceptives. Serum- and glucocorticoid-inducible kinase 1 (SGK) and proprotein convertase 5/6 (PC6) [149] are enzymes crucial to developing endometrial epithelial receptivity to implantation. Interestingly, a decline of SGK is required to enable endothelial receptivity, though higher levels are required for maintenance of a pregnancy, which makes commercial SGK-inhibitors better suited for infertility treatments rather than contraceptives [161]. Nona-D-arginine, or Poly-R, is a peptide-based inhibitor of PC6 which has shown the ability to prevent implantation. Additionally, targeting PC6 may have potential as an MPT because proprotein convertases have been implicated in the cellular processing of gp160 in HIV-1 and Poly-R has specifically shown inhibition of infection in vitro [150].

Other known implantation proteins with potential as contraceptive targets include cytokines LIF-6 and IL-11. LIF-6 binds to uterine epithelial proteins to form an activated complex involved in endometrial cell adhesion and stromal cell decidualization which are critical to embryo implantation. A PEGylated LIF-antagonist was shown to inhibit human embryo attachment in vitro, though LIF’s ubiquitous nature makes it difficult to target while avoiding off-target effects elsewhere in the body [151]. IL-11 has also been identified as a key cytokine for embryo implantation. A similar PEGylated-IL-11 antagonist, administered intraperitoneally and intravaginally in mice, reduced implantation site size by altering the formulation of decidual cells [152].

### 3.3. Immunocontraception

Anti-sperm antibodies can occur naturally, and while they may play a role in infertility, they can also be exploited to prevent unintended pregnancy [162]. Numerous essential sperm proteins have been identified and characterized as potential antigens for immunocontraception, including some previously discussed as targets for small molecule inhibition, like PH-20 [138], LDH-C4 [163], and IZUMO [164].

LDH-C4 is one of the earliest sperm antigens studied for efficacy in non-human primates [165]. The immunodominant B-cell epitope of the enzyme was chimerized with a stimulating tetanus T-20 cell epitope and used to elicit immunogenic responses in baboons. Unfortunately, only a 62% reduction in fertility was observed [143].

The sperm-surface hyaluronidase PH-20, also known as SPAM-1, facilitates several critical processes during fertilization, including zonal penetration [138]. It is highly conserved among mammalian models of human contraception such as rodents, bovines, and monkeys, potentially streamlining clinical translation. In male guinea pigs, immunization with as low as 5 µg (1.5 µg per kg) injection of PH-20 provided a complete and reversible loss of fertility by generating an anti-sperm immune response [139,166]. Female macaques immunized with portions of PH-20, truncated to reduce bacterial toxicity related to the full-length glycoprotein, also displayed significant immune responses, suggesting that PH-20 may be further explored for both male and female contraceptive vaccines [140].

Sperm–egg fusion is partially facilitated by the pairing of transmembrane sperm protein Izumo1 with the oocyte receptor, Juno. Loss-of-function studies have shown that knock-out of only one of these proteins is necessary to inhibit successful gamete fusion and fertilization [135]. In vitro, anti-Izumo antibodies were capable of reducing sperm–egg fusion events, though no effects on fertility were observed in immunized mice, male or female [136].

Additional sperm-specific targets continue to be established for female immunocontraception, though most have not achieved desired levels of fertility reduction in vivo. While immunization of female mice with sperm-specific antigens YLP-12 [167], SP-56 [168], P10G [169], SACA-3 [170], and FA-1 [171,172] have demonstrated reductions in number of litters and litter size, no complete contraceptive effect has been observed.

Recombinant TSA-1 [173] and SP-10 [174] were capable of producing high antibody titers when dosed to female rabbits and macaques, respectively, but studies investigating the efficacy of the antibodies to prevent pregnancy in vivo have not been performed.

Surprisingly, few studies have been conducted on immunizing male mammals with sperm-specific antigens. A synthetic peptide of sperm flagella protein-2 (SFP-2) reversibly reduced fertility by 80% in male mice [175]. Additionally, a synthetic peptide of the Human Sperm Antigen (HSA) N-terminus was optimized in male rabbits and subsequently used to immunize male marmosets. The peptide induced high antibody titers in seven out of nine marmosets, and all but one experienced infertility [176].

Continued research has established several additional sperm-specific targets, including SAMP14 [144], SAMP32 [145], ESP [146], AKAP3/4 [177], and CD46 [148,178], that are necessary for acrosomal reaction reactiveness. AKAP3/4 and CD46 have been determined as potential targets affecting sperm quality through preliminary research in protein disruption [147,148]. ESP, localized on the surface of sperm between the acrosome and post-acrosomal sheath, has also been demonstrated to play an essential role in fertilization through hamster egg penetration assays [146]. Sperm acrosomal membrane protein (SAMP) 14 and 32 antibodies have shown inhibition of fertilization in vitro, although further in vivo studies are required.

Zona pellucida sperm-binding protein-3, ZP3, is a glycoprotein responsible for the formation of the extracellular matrix coating that protects oocytes and facilities fertilization, making it a unique instance of an oocyte-specific contraceptive target [179]. Genetic studies have shown that ZP3 is necessary for mouse oocyte function and fertilization [180]. Side-effects of ZP3 genetic knock-out were not observed in somatic tissues, making ZP3 an especially attractive target for safe contraception [181]. Immunization with peptide mimics and recombinant proteins of ZP3 have resulted in antibody induction and reductions in fertility in several female animal models [141,180,182,183]. One pilot study in eastern grey kangaroos achieved complete infertility upon immunization with recombinant ZP3 in Freund’s complete adjuvant [142]. These results suggest that additional factors may need to be considered to develop a successful immunocontraceptive.

Strategies to enhance immunocontraceptive effects include targeting several proteins at once by vaccination with multiple peptides, as well as direct administration of antibodies. The sperm antigen Sp17 achieved limited efficacy alone, but increases in antibody titer were observed when a SP17 fragment was combined with tandem copies of Gonadotropin-Release Hormone (GnRH) in a fusion protein [184,185]. Though not entirely non-hormonal, this immunocontraceptive technique allowed for nearly 90% of female mice immunized with the fusion protein to experience infertility [185]. Most recently, a chimeric recombinant protein was developed with epitopes for Izumo, SACA-3, and PH-20, targeting gamete adhesion and fusion. Complete infertility was induced in 80% of immunized female mice, and the remaining 20% experienced a near 50% reduction in litter size [186].

CD52g, a glycoprotein, is anchored to the sperm plasma membrane and is the target of monoclonal antibody H6-3C4, cloned from a woman with infertility [187]. A human mAb IgG of this antibody has high specificity for CD52g. In vitro studies have shown the antibody’s ability to immobilize human sperm. Highly multivalent versions of H6-3C4 have been engineered with additional Fabs to enhance the speed of sperm agglutination, or clumping [188]. Further, they required a ten-fold lower dose than the parent IgG to efficiently reduce human sperm motility in the sheep vagina. Since CD52g is only present on human and chimpanzee sperm, direct effects of these antibodies on fertility cannot be studied in typical animal models, but results from an ongoing clinical trial (NCT04731818) with monovalent anti-CD52g IgG are expected to elucidate their true contraceptive potential.

### 3.4. Non-Surgical Alternatives for Permanent Sterilization

Although surgical sterilization is already non-hormonal by design, new techniques for permanent contraception can help provide alternatives to match user preference. Existing sclerosing drugs have been repurposed for tubal occlusion by creating scar tissue within the fallopian tubes to prevent an ovum from reaching sperm [189].

The use of quinacrine pellets for sclerosive human female sterilization has been documented in the past [189], though poorly supported safety concerns halted the practice. Since, studies have disputed these suggestions and shown the high tolerability of quinacrine for permanent sterilization [189] More recently, polidocanol foam (PF) has been administered trans-cervically to cause fallopian sclerosis in non-human primates to prevent pregnancy, but complete sterilization required doses (5% PF) higher than those approved for clinical venous sclerotherapy (1% PF) [190,191,192].

Overall, these non-surgical alternatives require additional optimization to enhance safety and ease of administration to be clinically viable.

## 4. Conclusions

The contraceptive market is flush with hormonal options, but with over 100 million unintended pregnancies occurring each year and negative perceptions of hormonal contraceptive side-effects and safety, interest in non-hormonal alternatives has grown. Existing non-hormonal contraceptives are limited to surgical sterilization, chemical and physical barriers, the copper IUD, and traditional family planning methods. Thus, the only highly efficacious long-acting reversible non-hormonal contraceptive available is the copper IUD. Additionally, the extremely limited number of male contraceptives furthers the need to establish non-hormonal technologies for both sexes.

Non-hormonal contraceptives can target one or more critical processes related to human reproduction, including the development of gametes, transit of sperm, ovulation of oocytes, and fertilization. Scientific advances continue to identify numerous proteins essential to the different stages, and several have been targeted for potential contraceptive benefit, with varied success at inhibiting fertility. Many additional approaches are being developed to address the gap in non-hormonal contraception, with much promise in local delivery of new antimicrobials and immunocontraceptives, as they are now reaching clinical trials.

Future directions should place attention on reproductive-specific targets such as those directly inhibiting gamete maturation (e.g., HIPK4, WEE2) and transit (e.g., EPPIN, sperm-specific ion channels), as well as fertilization (e.g., IZUMO1, JUNO, PH-20, ZP3, CD52g) and implantation (e.g., LIF-6, IL-11) to reduce off-target and side effects like those prevalent with use of hormonal contraceptives. Continued research and optimization are needed to bring enough new non-hormonal contraceptives to fulfill every remaining unmet need and empower users to have complete control over reproductive desires.

## Figures and Tables

**Table 1:** Existing Non-Hormonal Contraceptives.

Method	Type	Duration	Efficacy ^1^
Tubal Ligation	Surgical Sterilization	Permanent	99.74% [50]
Vasectomy	Surgical Sterilization	Permanent	99% [51]
Copper IUD	-	Long-Acting	99.2%, 99.9% ^2^ [52,53]
Condom	Physical Barrier	Short-Acting	87.0% [11]
N-9	Chemical Barrier	Short-Acting	78–90% [54]
Phexxi	Chemical Barrier	Short-Acting	86% [55]
Withdrawal	Traditional	-	80% [56]
Traditional Family Planning	Traditional	-	79.6–96.2% [57]

^1^ with typical use, ^2^ as emergency contraception.

**Table 2:** Non-Hormonal Reproductive Targets and Drug Candidates in Development.

Affected Process	Target	Drug Candidate
Spermatogenesis	BRDT	JQ1 [108,109,110]
	RARα	YCT529 [111]
	HIPK4	In Development [112]
	TSSK	In Development [113]
	Inter-Sertoli Junctions	Oleanolic Acid [114,115,116,117]
	CBR2	β-caryophyllene [118]
	Sertoli–Germ Cell Junctions	Triptonide [119]
		Adjudin [120]
		H2-Gamendazole [121]
Oogenesis	PDE3	Milrinone [122]
		ORG20864 [123]
Liquefaction	EPPIN	EP055 [124]
	PSA	AEBSF [125]
Capacitation	Na,K-ATPase 4	In Development [126]
	KSper	In Development [127]
	CatSper	HC-056456 [128]
	sAC	In Development [129]
Ovulation	COX-2	Celebrex [130]
		Meloxicam [130]
		BAY06 [131]
	ABCC4	In Development [132]
Fertilization	WEE2	In Development [133,134]
	JUNO	In Development [135]
	IZUMO1	Biologic [136]
	TMEM95	In Development [137]
	PH-20	Biologic [138,139,140]
	ZP3	Biologic [141,142]
	LDH-C4	Biologic [143]
Acrosome Reaction	SAMP14	Biologic [144]
	SAMP32	Biologic [145]
	ESP	In Development [146]
	AKAP3/4	In Development [147]
	CD46	In Development [148]
Implantation	SGK	In Development [149]
	PC6	Poly-R [150]
	LIF-6	In Development [151]
	IL-11	In Development [152]

## Data Availability

Not applicable.
